# Lung cancer prediction using machine learning on data from a symptom e-questionnaire for never smokers, formers smokers and current smokers

**DOI:** 10.1371/journal.pone.0276703

**Published:** 2022-10-21

**Authors:** Elinor Nemlander, Andreas Rosenblad, Eliya Abedi, Simon Ekman, Jan Hasselström, Lars E. Eriksson, Axel C. Carlsson

**Affiliations:** 1 Division of Family Medicine and Primary Care, Department of Neurobiology, Care Sciences and Society, Karolinska Institutet, Solna, Sweden; 2 Academic Primary Health Care Centre, Region Stockholm, Stockholm, Sweden; 3 Regional Cancer Centre Stockholm-Gotland, Region Stockholm, Stockholm, Sweden; 4 Division of Clinical Diabetology and Metabolism, Department of Medical Sciences, Uppsala University, Uppsala, Sweden; 5 Thoracic Oncology Centre, Karolinska University Hospital, Dept of Oncology-Pathology, Karolinska Institutet, Stockholm, Sweden; 6 Division of Nursing, Department of Neurobiology, Care Sciences and Society, Karolinska Institutet, Huddinge, Sweden; 7 School of Health and Psychological Sciences, City, University of London, London, United Kingdom; 8 Medical Unit Infectious Diseases, Karolinska University Hospital, Huddinge, Sweden; University of Bonab, ISLAMIC REPUBLIC OF IRAN

## Abstract

**Purpose:**

The aim of the present study was to investigate the predictive ability for lung cancer of symptoms reported in an adaptive e-questionnaire, separately for never smokers, former smokers, and current smokers.

**Patients and methods:**

Consecutive patients referred for suspected lung cancer were recruited between September 2014 and November 2015 from the lung clinic at the Karolinska University Hospital, Stockholm, Sweden. A total of 504 patients were later diagnosed with lung cancer (n = 310) or no cancer (n = 194). All participants answered an adaptive e-questionnaire with a maximum of 342 items, covering background variables and symptoms/sensations suspected to be associated with lung cancer. Stochastic gradient boosting, stratified on smoking status, was used to train and test a model for predicting the presence of lung cancer.

**Results:**

Among never smokers, 17 predictors contributed to predicting lung cancer with 82% of the patients being correctly classified, compared with 26 predictors with an accuracy of 77% among current smokers and 36 predictors with an accuracy of 63% among former smokers. Age, sex, and education level were the most important predictors in all models.

**Conclusion:**

Methods or tools to assess the likelihood of lung cancer based on smoking status and to prioritize investigative and treatment measures among all patients seeking care with diffuse symptoms are much needed. Our study presents risk assessment models for patients with different smoking status that may be developed into clinical risk assessment tools that can help clinicians in assessing a patient’s risk of having lung cancer.

## 1. Introduction

Lung cancer is globally the second most commonly diagnosed cancer with over 2,2 million new cases and the leading cause of cancer death, with an estimated 1.8 million deaths in 2020 [1]. Based on figures for 2019 from the Global Burden of Disease, the incidence of tracheal, bronchus and lung-cancer was reported to be 29.2, (Globally), 68.9 (Western Europe) and 42.5 (Sweden) cases per 100 000 inhabitants. Corresponding figures of mortality were 26.4, 59.8 and 42.4 respectively [2]. Fortunately, the most important risk factor for lung cancer, smoking [3], is declining in most Western countries [4]. With only 16% smokers, Sweden has the lowest prevalence in Europe, half of the European average of 27% [4]. Never smokers thus constitutes an increasing part of lung cancer patients in Sweden [3].

While early detection is crucial for prognosis, early symptoms and signs of lung cancer are often non-specific and common [5–7]. This is challenging for general practitioners (GPs), who must assess the likelihood of lung cancer and prioritize investigations and treatments among large groups of patients with non-specific and common symptoms, as for example fatigue or cough. Methods for assessing patients’ likelihood for cancer prior to the investigation of various symptoms and signs that may raise cancer suspicion are lacking. Risk assessment tools (RATs) for cancer, i.e., tools that translate epidemiological risk factors to applicable individual patient assessments, are lacking in primary health care (PHC).

Despite several studies conducted on various tools that assess patients’ cancer risk based on symptom presentation [8–10], there is insufficient evidence that cancer RATs affects the clinical outcome and more research is recommended in a recent health technology assessment conducted 2020 [11]. Patients in different health care systems should be studied due to variations in both risk factors, symptom presentation and documentation. The Patient EXperience of Bodily Changes for Lung Cancer Investigation (PEX-LC) study has published a model for predicting lung cancer based on reported symptoms and signs among patients having undergone PHC investigation [12]. PEX-LC has not yet been stratified on smoking status or applied to an unfiltered PHC population [12]. The rich material of the PEX-LC study provides a starting point for further studies in a PHC context. The aim of the present study was to investigate the predictive ability for lung cancer of symptoms reported in the PEX-LC study, separately for never smokers, former smokers, and current smokers, to create models for future testing in a PHC population.

## 2. Methods

### 2.1 Study design

**Fig 1:**
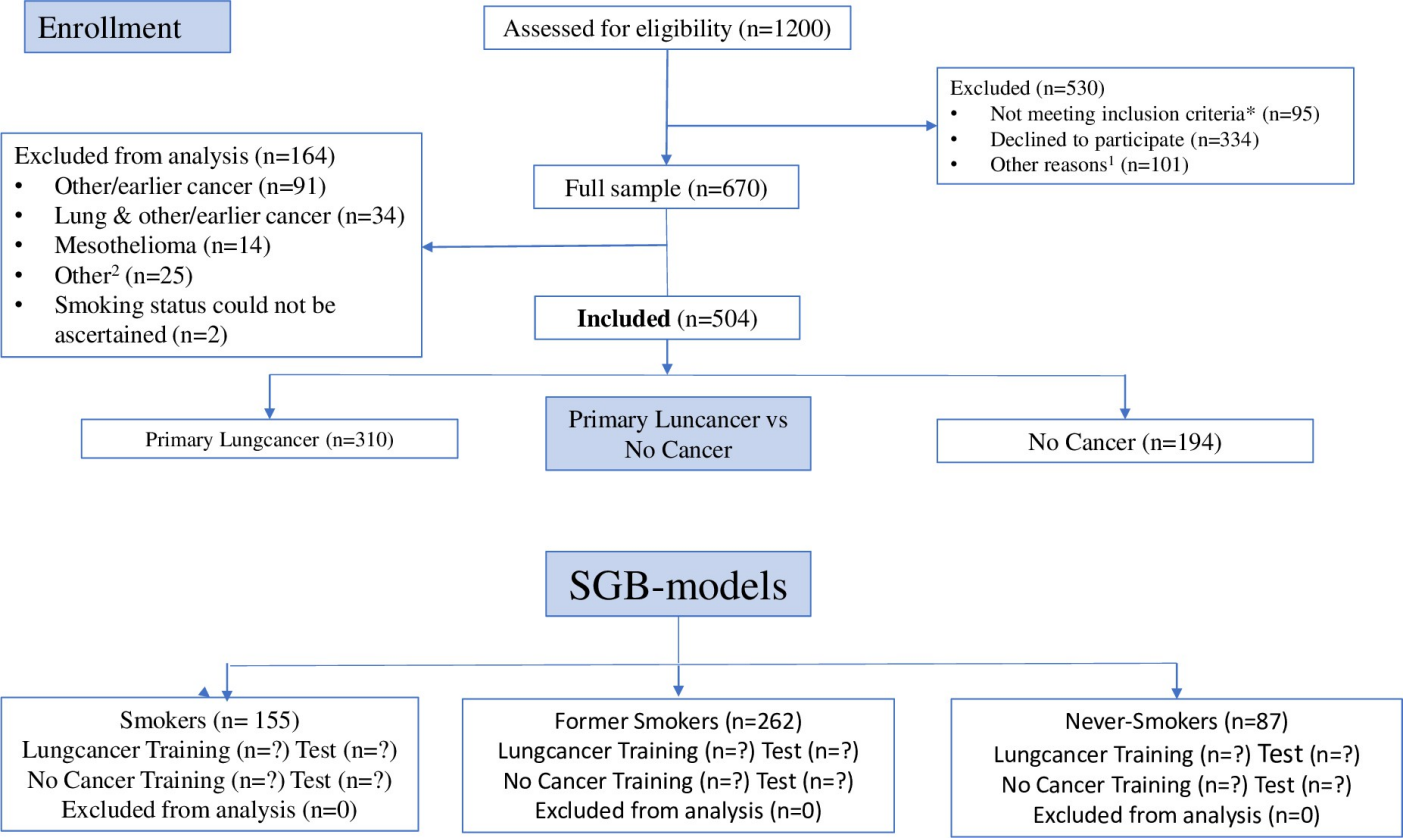
CONSORT flow diagram: The PEX-LC lung cancer investigation cohort. This figure is based on the CONSORT 2010 flow diagram. As this was not a randomised intervention trial, it has been modified to suit this cohort study accordingly. Primary lung cancer (no other cancer); NSCLC: non-small cell lung cancer (adenocarcinoma, n = 200; squamous cell carcinoma, n = 45; not otherwise specified (NOS), n = 5; other NSCLC (adenosquamous lung carcinoma (n = 4), large cell neuroendocrine carcinoma (n = 3); large cell carcinoma, adenoid cystic carcinoma of the lung, adenoid carcinoma with neuroendocrine differentiation, and mucoepidermoid carcinoma of the lung (n = 1, respectively)); SCLC: Small cell lung cancer (includes one individual with combined SCLC) (n = 24); Other LC: carcinoid, n = 9; no histology, n = 17. * Not meeting inclusion criteria: translator required (n = 50), consent withdrawn/missing (n = 15); missing data (n = 5); other reason such as or pain, illness, or other medical condition (n = 25). ^1^ Other reasons: Limited time of the visit or lack of resources (staff) at the clinic (n = 47); hospitalisations (n = 34); deaths (n = 20). ^2^ Other: Medical records non-consent (n = 4); unconfirmed, possible lung cancer (n = 3); undiagnosed cancer (n = 2); death before clinical investigation (n = 1); participant withdrew clinical investigation (n = 2); previous lung cancer (n = 1); incomplete modules (n = 12).

Participants were recruited among 1200 consecutive patients referred for suspected lung cancer between September 2014 and November 2015 to the lung clinic at the Karolinska University Hospital, Stockholm, Sweden [12]. Of the 670 patients agreeing to participate, 506 patients were later diagnosed with either lung cancer or no cancer. The remaining 164 patients were excluded due to multiple other diagnoses (primarily previous cancer, or a cancer diagnosis other than lung cancer). Additionally, for the present study, two patients whose smoking status could not be ascertained were excluded, resulting in a study sample of 504 patients, of which 310 (61.5%) were diagnosed with lung cancer and the remaining 194 patients (38.5%)with no cancer, see CONSORT flow diagram in Fig 1.

### 2.2. Questionnaire

Participants completed the PEX-LC adaptive e-questionnaire on a touch screen before their clinical visit. Research assistants were available for help. The number of questions each patient answered differed depending on their symptoms and sensations, with a maximum of 342 potential items: 285 descriptors indicative of the first symptoms/sensations the patient noticed that had caused a change in their lives, and 57 background variables. Medical records of eventual diagnosis were later retrieved, with a follow-up after questionnaire completion of ≥ 1 year.

The questionnaire has been described in detail elsewhere [12, 13]. In short, PEX-LC was tailored to allow participants to complete only those items appropriate for the individual’s onset of symptoms or sensations. Background variables included socio-demographics, comorbidities, and smoking habits. Symptoms and sensations included breathing difficulties, cough, phlegm/expectorates, pain/aches/discomfort, fatigue, voice changes, appetite/eating/taste changes, olfactory changes, and fever/chills/sweating. Finally, other changes were also included, for example general physical condition, malaise, or other emotional changes.

### 2.3. Smoking status

Smoking status was assessed by asking about current and former smoking habits, as well as recent changes in smoking habits. Based on this, participants were classified as never smokers (smoked < 100 cigarettes in their lifetime), former smokers (daily smokers that quit during the year before commencement in the study), or current smokers. Participants having “other smoking habits” could describe these in free text and based on this were classified into one of the three groups never smokers, former smokers, or current smokers, or denoted as having a missing value for this variable.

### 2.4. Statistical analyses

All analyses were performed separately for the three groups never smokers, former smokers, and current smokers. Categorical data are presented as frequencies and percentages, n (%), while continuous data are given as means with accompanying standard deviations (SDs). Tests of differences between groups were performed using Pearson’s χ^2^-test for categorical data and one-way ANOVA for continuous data. Stochastic gradient boosting (SGB) [14], implemented in the R package ‘gbm’ version 2.1.8 [15], was used to predict if a patient had lung cancer or not. A training-test approach was applied to the data, whereby 70% of the observations were randomly selected for training the SGB model, which was then tested on the remaining 30% of the observations to evaluate its performance. The random selection of patients to include in the training data set was performed using stratification on later diagnosed lung cancer status (Lung cancer/Not lung cancer), to ensure equal proportions of lung cancer cases in the training and test data sets and enough cases in each subgroup. The SGB models used a Bernoulli loss function fitted to 10 000 trees, each having a maximum depth of 5 interactions, with a shrinkage (learning rate) of 0.001, a minimum of 10 observations in the terminal nodes of the trees, and a subsampling rate (bag fraction) of 0.5. The optimal number of trees to use for prediction was estimated using 10-fold cross validation.

Using these trees, the SGB models were applied to the training and test data sets to obtain the individual probabilities of having lung cancer for each patient. Cut-off values for classifying patients in the test data set as having lung cancer or not were then constructed by calculating the value of the percentile of these individual probabilities for the training data set that corresponded to the proportion of patients in the training data set that were known to not have lung cancer. A patient in the test data set was then classified as having lung cancer if the individual probability of having lung cancer obtained from the SGB model was larger than this cut-off value, and otherwise classified as not having lung cancer. The performance of the SGB models were evaluated using area under the receiver operator characteristic (ROC) curve (AUC), confusion matrixes, overall accuracy, sensitivity, specificity, positive predicted value, and negative predicted value [14, 16, 17]. Variable importance was estimated by normalized relative influence (NRI), were the relative influences are normalized to sum to 100 [18]. All statistical analyses were performed using R version 4.1.0 (R Foundation for Statistical Computing, Vienna, Austria), with two-sided P-values < 0.05 considered statistically significant.

### 2.5. Ethics

All patients gave their written informed consent to participate before their first scheduled visit. The study was carried out according to the Declaration of Helsinki and data were pseudonymized to protect the privacy of the participants. Approval was obtained from the Stockholm Regional Ethics Review Board (Dnr 2014/1290–32). Data are available upon reasonable request from prefekt@nvs.ki.se.

## 3. Results

### 3.1. Participant characteristics

Table 1 presents the characteristics of the 504 participants according to smoking status: 87 (17.3%) never smokers, 262 (52.0%) former smokers, and 155 (30.8%) current smokers. The participants were at a mean age of 68.3 years, with 50.6% (n = 255) being males and 83.5% (n = 421) being born in Sweden. About one of three (n = 181; 35.9%) participants had a college/university education, and six out of ten (n = 310; 61.5%) participants had lung cancer. The participants differed significantly regarding age (P < 0.001), education level (P = 0.021), and lung cancer status (P < 0.001), with never smokers being the youngest (mean age 68.3 years), having the highest proportion of participants with a college/university education (n = 42; 48.3%), and having the lowest prevalence of lung cancer (n = 33; 37.9%). Former smokers were the oldest (mean age 70.5 years), while current smokers had the lowest proportion of participants with a college/university education (n = 45; 29.0%) and the highest prevalence of lung cancer (n = 114; 73.5%).

**Table 1:** Characteristics of the 504 participants according to smoking status.

Variable	All (n = 504)	Never smoker (n = 87)	Former smoker (n = 262)	Current smoker (n = 155)	P-value
Age (years), mean (SD)	68.3 (10.8)	63.9 (13.5)	70.5 (9.9)	67.3 (9.7)	**< 0.001**
Male sex, n (%)	255 (50.6)	42 (48.3)	140 (53.4)	73 (47.1)	0.408
Born in Sweden, n (%)	421 (83.5)	70 (80.5)	221 (84.4)	130 (83.9)	0.692
Education level, n (%)					**0.021**
• Other	43 (8.5)	9 (10.3)	21 (8.0)	13 (8.4)	
• Primary school	165 (32.7)	15 (17.2)	89 (34.0)	61 (39.4)	
• Secondary school	115 (22.8)	21 (24.1)	58 (22.1)	36 (23.2)	
• College/University	181 (35.9)	42 (48.3)	94 (35.9)	45 (29.0)	
Lung cancer, n (%)	310 (61.5)	33 (37.9)	163 (62.2)	114 (73.5)	**< 0.001**

Notes: SD, standard deviation. Significant P-values are given in **bold**.

### 3.2. Performance of the SGB models

**Fig 2:**
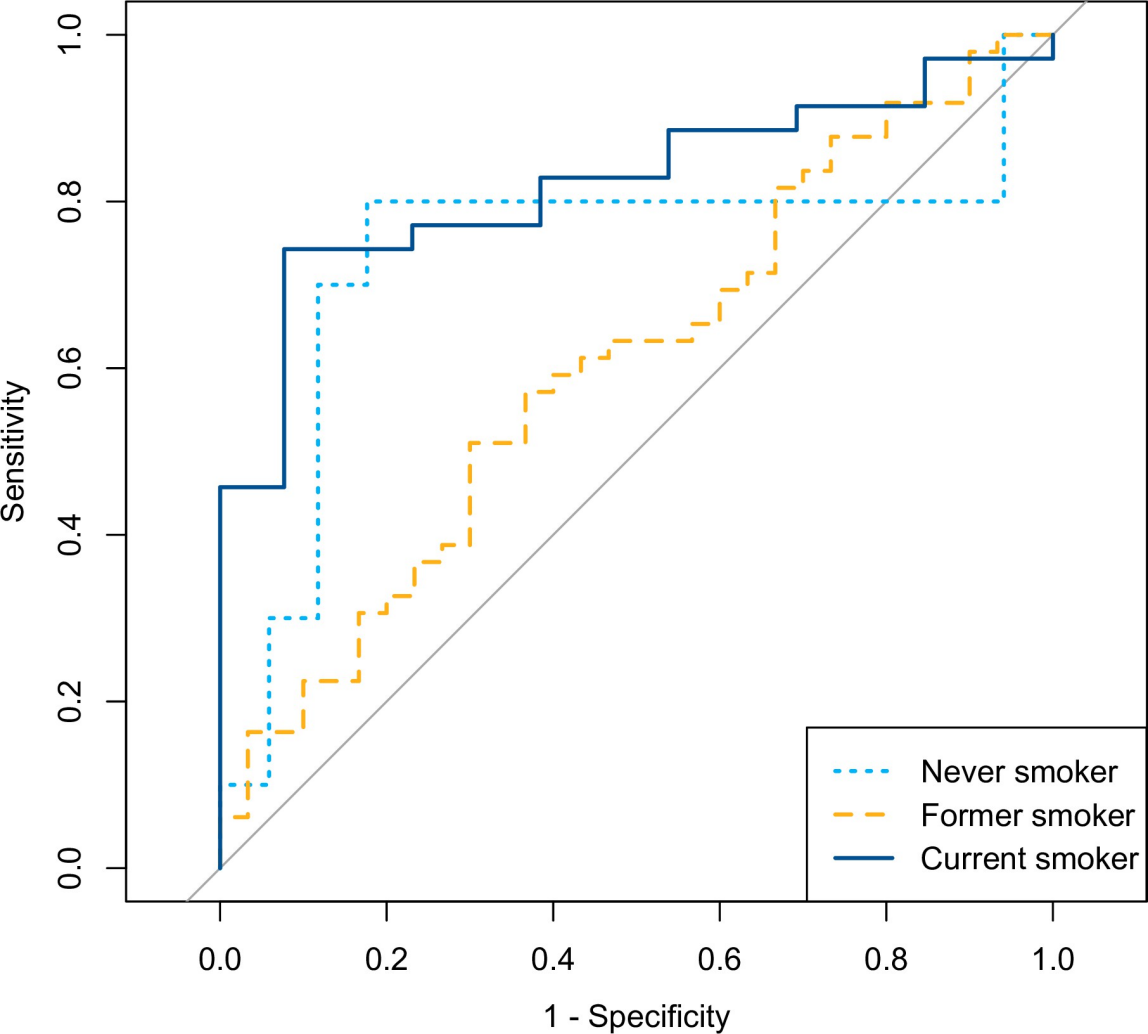
ROC curves for the three groups never smoker, former smoker, and current smoker.

Tables 2 and 3 present confusion matrixes and performance measures, respectively, for predictions of lung cancer status for patients in the test datasets using SGB models from the training datasets, according to smoking status. ROC curves for the three groups are given in Fig 2. The optimal number of trees to use for the predictions were 976 for never smokers, 1245 for former smokers, and 1472 for current smokers. Overall, the SGB models performed well for never smokers and current smokers, with AUC values of 0.735 and 0.822, respectively, and corresponding overall accuracies of 0.815 and 0.771. The performance was considerable worse for former smokers, with an AUC of 0.604 and an overall accuracy of 0.633. While the sensitivity was high for former and current smokers, with values of 0.816 and 0.829, respectively, the sensitivity of 0.700 for never smokers was low. The specificity of 0.882 for never smokers was, on the other hand, high, while former smokers had a very low specificity of 0.333.

**Table 2:** Confusion matrixes for predictions of lung cancer status for patients in the test data sets using stochastic gradient boosting models from the training data sets, according to smoking status.

		Observed		
Smoking status	Predicted	Lung cancer	Not lung cancer	Total
Never smoker^a^	Lung cancer	7	2	9
	Not lung cancer	3	15	18
	Total	10	17	27
Former smoker^b^	Lung cancer	40	20	60
	Not lung cancer	9	10	19
	Total	49	30	79
Current smoker^c^	Lung cancer	29	5	34
	Not lung cancer	6	8	14
	Total	35	13	48

Notes: Predictions based on ^a^ 976 trees; ^b^ 1245 trees; and ^c^ 1472 trees.

**Table 3:** Performance measures for predictions of lung cancer status for patients in the test data sets using stochastic gradient boosting models from the training data sets, according to smoking status.

Measure	Never smoker (n = 27)^a^	Former smoker (n = 79)^b^	Current smoker (n = 48)^c^
AUC	0.735	0.604	0.822
Overall accuracy	0.815	0.633	0.771
Sensitivity	0.700	0.816	0.829
Specificity	0.882	0.333	0.615
Positive predicted value	0.778	0.667	0.853
Negative predicted value	0.833	0.526	0.571

Notes: AUC, area under the ROC curve. Predictions based on ^a^ 976 trees; ^b^ 1245 trees; and ^c^ 1472 trees.

### 3.3. Variable importance

Of the 73 predictors included in the SGB models, 17 (23.3%) had a non-zero influence for never smokers, 36 (49.3%) for former smokers, and 26 (35.6%) for current smokers. The ten predictors with the highest NRI for the SGB models, according to smoking status, are given in Table 4. Age was the dominant predictor regardless of smoking status, accounting for 51.0% of the influence on the probability of being diagnosed with lung cancer among never smokers, 35.3% of the influence among current smokers, and 28.6% of the influence among former smokers, while education level and sex came in as the second and third most important predictor, respectively, with NRI values between 6 and 10 percent. Of the other variables, “Breathing worse upon exertion” was among the five most important predictors for all smoking groups, with NRI values > 5.0% for both never smokers and current smokers, while “Antibiotics within the past 2 years”, “Cough varied over the day”, “Voice got hoarser”, and “A cold, flu or pneumonia within the past 2 years” were all among the top ten predictors for all smoking groups, with NRI values between 1.9 and 5.7 percent. Notably, the predictor “Haemoptysis/hematemesis” (blood-mixed/brown sputum) had a non-zero influence for never smokers and smokers. For former smokers, “Haemoptysis/hematemesis”was ranked 26^th^ among the 36 predictors with non-zero influence, with an NRI of only 0.56%.

**Table 4:** Predictors with highest normalized relative influence (NRI) for the stochastic gradient boosting models according to smoking status.

	Never smoker^a^		Former smoker^b^		Current smoker^c^	
Rank	Predictor	NRI	Predictor	NRI	Predictor	NRI
1	Age	51.0	Age	28.6	Age	35.3
2	Education level	8.8	Education level	9.9	Education level	8.5
3	Sex	6.2	Sex	7.5	Sex	7.3
4	Antibiotics within the past 2 years	5.7	Antibiotics within the past 2 years	4.9	Breathing worse upon exertion	5.7
5	Breathing worse upon exertion	5.5	Breathing worse upon exertion	4.9	Appetite loss	4.8
6	Feeling unfit	5.0	Voice got hoarser	4.2	Antibiotics within the past 2 years	4.7
7	Cough varied over the day	4.8	Cough varied over the day	4.0	Less strength, got weaker	4.1
8	Felt cold	4.4	A cold, flu or pneumonia within the past 2 years	3.5	Cough varied over the day	3.9
9	Voice got hoarser	2.5	Wheezing/panting	3.0	Voice got hoarser	3.5
10	A cold, flu or pneumonia within the past 2 years	1.9	Feeling unfit	2.8	A cold, flu or pneumonia within the past 2 years	3.3

Notes: Of the 73 predictors

^a^ 17 (23.3%)

^b^ 36 (49.3%); and

^c^ 26 (35.6%) had non-zero influence.

## 4. Discussion

Among never smokers, 17 predictors contributed to predicting lung cancer with 82% of the patients being correctly classified, compared to 26 predictors with an accuracy of 77% for current smokers and 36 predictors with an accuracy of 63% for former smokers.

### 4.1. Results in perspective

Several large medical record-based cohort studies carried out in PHC and a prospective cohort study on both medical record data and questionnaires from patients referred to a lung cancer clinic have shown hemoptysis, dyspnea, chest pain, cough, appetite loss and/or weight loss to have predictive capability for lung cancer [5, 6, 19–22]. All these variables, except hemoptysis, were important predictors for lung cancer in our models.

Notably, the predictor “Haemoptysis/hematemesis” (blood-mixed/brown sputum), was identified as the most important predictor for lung cancer in a previous study of referred patients [21], but had in the present study a non-zero influence only for former smokers. However, whereas Walter et al. [21] recruited patients referred to respiratory clinics with any symptoms suspicious of lung cancer noted in the referral letters, with a response rate of 19.5%, our study recruited participants among 1200 consecutive patients referred to a secondary clinic explicitly for suspected lung cancer, with a response rate of 55.8%, making direct comparisons challenging. Furthermore, the incidence of lung cancer in our study was 62% compared with 19% in Walter et al. [21]. A study investigating changes in the presenting symptoms of lung cancer from 2000–2017 in the UK PHC found that patients with lung cancer presenting with symptoms of haemoptysis are now rare [23]. This is consistent with our results even though the patients in our study had already been referred to secondary care with suspected lung cancer and had already passed PHC assessment.

Chest pain had a non-zero influence in all models, with back pain having a non-zero influence also for current and former smokers. However, no model included chest or back pain among the ten variables with the highest importance. Besides age, sex and education level were the most important predictors. The same result was found, when the PEX-LC data were analyzed with smoking as a predictor [12]. Sex being a variable of such high importance was then conjectured to be due to a higher proportion of smokers among women, but in the present analysis, this result holds also for never smokers. Age and sex are also determining factors for treatment effects of lung cancer [24].

Previous studies from Korea and Sweden have suggested that the incidence of lung cancer in never smokers has increased [25, 26]. A Finnish study pooled seven cohorts and studied five risk factors for lung cancer in over 100 000 never smokers [27]. They found no general increase of lung cancer in never smokers, although the proportion of adenocarcinoma type of lung cancer among women had increased more sharply during the past 10 years. In contrast to the present study, education level was not predictive for lung cancer in the Finnish study, and height was the only factor associated with lung cancer. Regrettably, we did not have access to data on height in our study and could therefore not evaluate its importance.

### 4.2. Clinical implications

RATs are much needed in PHC settings. Current national clinical guidelines for lung cancer in Sweden give GPs in PHC little support in finding lung cancer, especially among never smokers. Investigation with chest X-ray or low-dose computerized tomography (CT)-scan is recommended for patients having haemoptysis or chest/shoulder pains without other explanations or if a smoking or former smoking patient coughs or has dyspnoea for > 6 weeks. Our prediction model for never smokers ranked chest pain 15^th^ among 17 predictors with non-zero influence, with an NRI of only 0.36%. This indicates that chest pain is a less useful predictor for patients who have been referred with suspected lung cancer to secondary care. Whether chest pain as a predictor have a higher influence in PHC should be further investigated.

Lung cancer diagnosis via symptoms and signs are sometimes downgraded in importance compared with screening. However, screening programmes for lung cancer have mostly been targeting high-risk smoking individuals [28], leaving the increasing group of never smokers without structured guidelines for early detection. Moreover, screening programs have partial uptake and limited sensitivity, and cancers occurring outside the screening age groups must be detected via symptoms and signs. Sweden do not currently have a screening program for lung cancer.

### 4.3. Limitations and strengths

Limitations of the present study include potential recall bias. Some of the questions in the e-questionnaire could have been more precise. Former smokers are a heterogeneous group including individuals who only smoked sporadically for a short period of time as well as patients who have been heavy smokers but quit > 1 year ago, which should contribute to the obtained model having a lower predictive ability for lung cancer in this group. Adding a question of package years would have facilitated the possibility of subgrouping. The e-questionnaire also lacked systematic questions about non-cigarette forms of tobacco and nicotine use. Due to the stratification according to smoking status and the resulting small sample sizes, the predictive value of rare-occurring descriptors may be underestimated due to chance. Since our study included patients referred from PHC for suspected lung cancer, the usefulness of the prediction models in a general PHC population remains to be investigated.

## 5. Conclusions

Tools assessing the likelihood of having lung cancer among patients with diffuse symptoms are much needed. This is especially true for never smokers who are often detected in a late stage. Our study presents risk assessment models that may be developed into clinical RATs that can help clinicians in assessing a patient’s risk of lung cancer. We welcome future studies conducted in PHC settings on assessable background variables and symptom combinations and their ability to predict lung cancer in patients with different smoking statuses.

## Abbreviations

CI: confidence interval
GP: general practitioner
NRI: normalized relative influence
PHC: primary health care
RAT: risk assessment tool
SD: standard deviation
SGB: stochastic gradient boosting
