# Correction: Lung cancer prediction using machine learning on data from a symptom e-questionnaire for never smokers, formers smokers and current smokers

**DOI:** 10.1371/journal.pone.0295780

**Published:** 2023-12-07

**Authors:** Elinor Nemlander, Andreas Rosenblad, Eliya Abedi, Simon Ekman, Jan Hasselström, Lars E. Eriksson, Axel C. Carlsson

Fig 1 is incorrect. The authors have provided a corrected version here.

**Fig 1:**
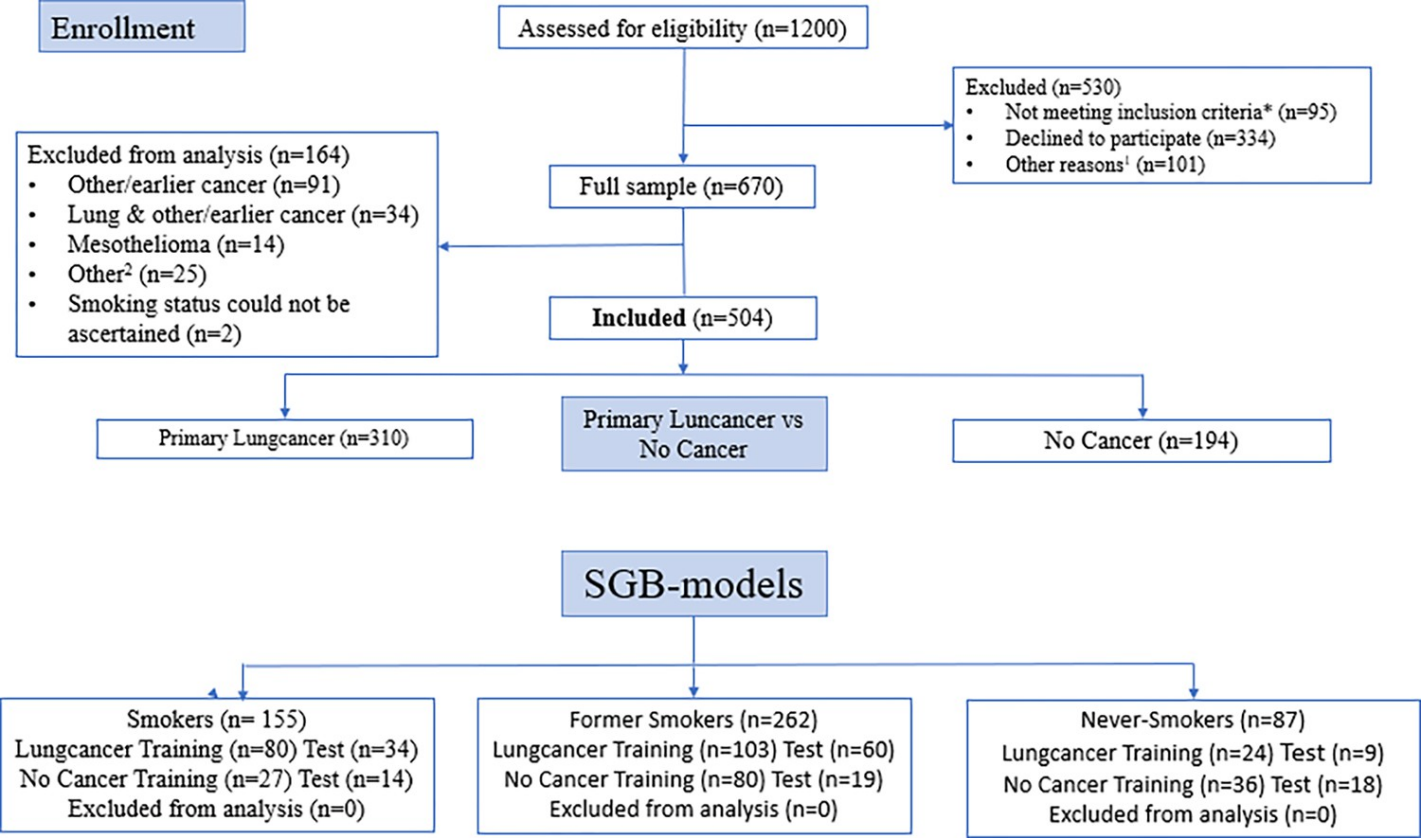
CONSORT flow diagram: The PEX-LC lung cancer investigation cohort. This figure is based on the CONSORT 2010 flow diagram. As this was not a randomised intervention trial, it has been modified to suit this cohort study accordingly. Primary lung cancer (no other cancer); NSCLC: non-small cell lung cancer (adenocarcinoma, n = 200; squamous cell carcinoma, n = 45; not otherwise specified (NOS), n = 5; other NSCLC (adenosquamous lung carcinoma (n = 4), large cell neuroendocrine carcinoma (n = 3); large cell carcinoma, adenoid cystic carcinoma of the lung, adenoid carcinoma with neuroendocrine differentiation, and mucoepidermoid carcinoma of the lung (n = 1, respectively)); SCLC: Small cell lung cancer (includes one individual with combined SCLC) (n = 24); Other LC: carcinoid, n = 9; no histology, n = 17. * Not meeting inclusion criteria: translator required (n = 50), consent withdrawn/missing (n = 15); missing data (n = 5); other reason such as or pain, illness, or other medical condition (n = 25). ^1^ Other reasons: Limited time of the visit or lack of resources (staff) at the clinic (n = 47); hospitalisations (n = 34); deaths (n = 20). ^2^ Other: Medical records non-consent (n = 4); unconfirmed, possible lung cancer (n = 3); undiagnosed cancer (n = 2); death before clinical investigation (n = 1); participant withdrew clinical investigation (n = 2); previous lung cancer (n = 1); incomplete modules (n = 12).
